# Neonatal Sepsis: A Comprehensive Review

**DOI:** 10.3390/antibiotics14010006

**Published:** 2024-12-25

**Authors:** Charikleia Kariniotaki, Christina Thomou, Despoina Gkentzi, Eleftherios Panteris, Gabriel Dimitriou, Eleftheria Hatzidaki

**Affiliations:** 1 Department of Neonatology/Neonatal Intensive Care Unit, School of Medicine, University of Crete, University Hospital of Heraklion, 71003 Heraklion, Greece; charikliakari@gmail.com (C.K.); cthomou@yahoo.gr (C.T.); eleftherios.panteris@gmail.com (E.P.); 2 Department of Paediatrics, University General Hospital of Patras, Patras Medical School, 26504 Rio, Greece; gkentzid@upatras.gr (D.G.); gdim@upatras.gr (G.D.)

**Keywords:** early-onset sepsis, late-onset sepsis, sepsis risk assessment tools, sepsis biomarkers

## Abstract

Neonatal sepsis remains a significant cause of neonatal morbidity and mortality globally. At present, no clear consensus definition for sepsis in neonates exists, even though a positive blood culture is considered as the gold standard for definitive diagnosis. The accurate and timely diagnosis of sepsis in neonates presents significant difficulties, since “culture negative” or “suspected” sepsis varies widely worldwide. Antibiotic overuse and resistance are emerging problems, but on the other hand, under-recognition of sepsis and delayed antibiotic treatment could have serious or even fatal adverse outcomes in this vulnerable population. In the context of rapid recognition of sepsis and timely initiation of appropriate antibiotic therapy, various sepsis risk assessment tools have been developed, a variety of biomarkers are in clinical use or under research for future use, and new diagnostic techniques are under evaluation. In this review, we summarize the most recent data on neonatal sepsis, the utility of sepsis risk assessment tools for term and preterm infants with sepsis, and current diagnostic and preventive tools.

## 1. Introduction

Neonatal sepsis is a significant cause of neonatal morbidity and mortality, with an incidence ranging from 1–4/1000 live births in the United States and significant variability worldwide. It is a clinical syndrome with symptomatology and laboratory findings consistent with a systemic inflammatory response to a multimodal infection from an array of hosts ranging from bacteria to viruses or fungi, potentially leading to multiorgan dysfunction, failure, and even death within the first 28 days of life [1,2].

A patient has “suspected” sepsis in the presence of clinical signs without laboratory evidence of infection (i.e., negative culture) and treatment for sepsis empirically with antibiotics for at least five days [3]. Confirmed or “culture-positive” sepsis patients have a positive culture from a typically sterile body fluid in addition to sepsis clinical symptoms [4]. The microorganism responsible may be isolated from the blood, urine, cerebrospinal fluid (CSF), peritoneal fluid, or other sterile tissue [4]. Despite common misconceptions, and even though a positive blood culture is considered the gold standard for the diagnosis, this condition may or may not be associated with bacteremia [5,6]. Bloodstream infections are considered a subgroup of neonatal sepsis, when a pathogen, from a symptomatic patient, is cultured from blood or cerebrospinal fluid. Meningitis in neonates is usually hematogenous. Thus, a positive CSF culture can be regarded as evidence of bloodstream infection regardless of blood culture results [3]. Septic shock is a severe form of sepsis with persistent hypotension, below the fifth percentile for age, that does not improve after adequate fluid resuscitation and requires urgent management with vasopressor agents to hemodynamic stabilization [7].

Despite advances in molecular diagnostics, the accurate and timely diagnosis of sepsis in neonates presents significant difficulties. The gold standard sepsis diagnosis method based on the presence of a positive culture does not rule out sepsis, since reported rates of “culture-negative” or “suspected” sepsis vary widely in the literature. Antibiotic overuse and resistance are emerging problems, while under-recognition of sepsis and delayed antibiotic treatment could have serious or even fatal outcomes on the neonatal population. Therefore, timely sepsis diagnosis and initiation of appropriate antibiotic therapy are of great importance. Considering the variability in the definitions of sepsis used in the literature [7], causing ambiguity in the interpretation, comparison, and generalization of the results, it is crucial to establish a clear consensus definition that differentiates sepsis from infection and bacteremia and therefore entails appropriate management of the patients [7,8,9].

In this review, the most recent data on neonatal sepsis are summarized, including its definition, pathophysiology, risk factors, and clinical presentation. Additionally, the utility of sepsis risk assessment tools for term and preterm infants is discussed, along with optimal antibiotic treatment strategies and potential modes of prevention.

## 2. Definition

Neonatal sepsis is classified, depending on the age of onset, as early-onset sepsis (EOS) and late-onset sepsis (LOS). EOS is defined by the Centers for Disease Control and Prevention (CDC) as an infection occurring in the newborn in the first seven days of life, whereas for infants hospitalized since birth, EOS is defined as an infection occurring within the first 72 h of age, since epidemiological studies have shown that beyond the first three days of life, nosocomial pathogens take over perinatally acquired infections [10]. LOS presents beyond 3 to 7 days of life due to organisms acquired from the interaction with the hospital environment or the community or, in some cases, due to parturition acquired organisms, but with clinical presentation of infection after 72 h of life [1]. This classification is essential because of each group’s different etiologic agents, modes of transmission, risk factors, evaluation, and management.

## 3. Pathogens and Modes of Transmission

### 3.1. Early-Onset Sepsis (EOS)

The most common pathogen of EOS, globally, is Group B *Streptococcus* (G.B. *Strep*.), prevailing in term infants, even though the incidence has decreased considerably after the widespread adoption of universal antenatal screening for vaginal and rectal G.B. *Strep* colonization at 35–37 weeks of gestation or at the time of premature rupture of membranes and the introduction of intrapartum antibiotic prophylaxis (IAP) with penicillin or ampicillin for colonized women [11,12]. This preventive strategy led to a >85% reduction in EOS secondary to G.B. *Strep*. [11,12]. The second most common causative agent is *Escherichia coli* (*E. coli*), which predominates in preterm infants, especially very low birth weight (VLBW) ones [10]. Group B Streptococcus (G.B. *Strep*.) and *E. coli* account for two-thirds of all cases of EOS. Other less common microorganisms are *Listeria monocytogenes*, *Staphylococcus aureus*, enterococci, other Gram-negative bacteria (*Klebsiella*, *Citrobacter*, *Serratia*, *Enterobacter*), anaerobes (especially *Bacteroides fragilis*), *Haemophilus influenzae*, *Streptococcus pneumoniae*, and *Candida* spp. These microorganisms are typically maternal genitourinary and gastrointestinal tract colonizers [10].

Intra-uterine infections occur more commonly from vertically transmitted ascending bacteria, mainly from the vaginal environment, that enter the amniotic fluid after premature rupture of membranes or during passage through the birth canal in vaginal delivery. Transplacental hematogenous transmissions occur less commonly, and even rarer is the transmission of retroperitoneal infections to the fetus through the fallopian tubes [1]. Chorioamnionitis or intraamniotic infection (IAI) refers to an acute inflammation of the fetal membranes due to a bacterial infection, often as a result of prolonged chorioamniotic membrane rupture. It presents with either symptoms from the mother (fever, leucocytosis, cloudy or odorous discharge, lower abdominal tenderness), from the fetus (tachycardia), or with laboratory or pathological abnormalities of the placenta (histological chorioamnionitis). Low gestational age and prolonged rupture of membranes are related to higher rates of chorioamnionitis, with *Ureaplasma* spp. being the most common pathogen isolated from the placenta and amniotic fluid [13]. It should be noted, however, that a recent systematic review and meta-analysis evaluating the contemporary bacterial pathogens of EOS (and LOS) revealed Gram-negative bacteria to be the predominant cause of both EOS and LOS in low- and lower-middle-income countries (LLMICs) [14].

### 3.2. Late-Onset Sepsis (LOS)

LOS is usually caused by horizontally transmitted environmental organisms, either nosocomial or community-originated. Venous catheters and other indwelling invasive medical devices, nutritional devices, and environmental surfaces are considered as possible sources of pathogen exposure, as well as contact with healthcare providers and family members [15]. Colonization of the fetus by vertically transmitted pathogens (mainly G.B. *Strep*.) can also result in LOS, albeit rarely.

The most commonly isolated pathogens in LOS are Gram-positive bacteria in high-income countries and Gram-negative bacteria in LLMICs [14,15]. The majority of Gram-positive bacteria, mainly coagulase-negative staphylococci (CoNS) and streptococci, cause clinically significant infection in preterm infants but are considered skin commensals in term neonates and should be discriminated from potential culture contamination [16,17]. Other Gram-positive bacteria associated with LOS are *Staphylococcus aureus*, *Enterococcus* spp., and G.B. *Strep*. Interestingly, late-onset G.B. *Strep* infection rates have remained relatively stable over the past 20 years and are not impacted by IAP [18]. More severe cases of LOS, with higher mortality rates, are usually attributed to Gram-negative microorganisms, with *E. coli* being the main representative. Other Gram-negative bacteria, isolated in hospitalized neonates, include *Klebsiella*, *Enterobacter*, *Citrobacter* spp., *Serratia*, *Acinetobacter*, and *Pseudomonas aeruginosa* [1,15]. The significant rise in *Klebsiella* spp. as dominant species in LOS (and EOS) has been reported in many recent epidemiological studies [14,19,20,21,22]. The rise of extended-spectrum beta-lactamase (ESBL)-producing *Enterobacteriaceae* in the recent decades, as well as carbapenemase-producing *Enterobacteriaceae* (CPE), is a global threat that perplexes LOS cases and antimicrobial treatment selection [23]. Most often, meningitis is a late-onset infection resulting from a microorganism’s hematogenous spread into the central nervous system (CNS) and less frequently results from a contiguous spread due to contamination of congenital defects of CNS or CNS devices.

Fungi, most frequently *Candida* spp., are implicated as causes of invasive infections during prolonged hospitalization of preterm neonates and should be suspected in ill-looking newborns with suspicious symptoms (e.g., rash, neutropenia, thrombocytopenia, hypoglycemia). *Candida* spp. are the third most common cause of LOS in VLBW infants, with *Candida parapsilosis* emerging as a major pathogen in neonates with central venous catheters [24]. *Candida auris* (*C. auris*) is an emerging fungal infection in at-risk populations such as extremely preterm infants exposed to broad-spectrum antibiotics and invasive medical procedures with very poor outcomes [25]. In more recent years, *C. auris* has caused outbreaks in NICUs and has subsequently become endemic in many hospitals in South Africa [26].

Infection with *Candida* spp. is associated with significant morbidity and mortality in infants. Extremely low birth weight (<1000 g) infants carry the highest burden of disease. Multiple risk factors associated with *Candida* spp. infection in neonates have been identified, such as gestational age, birth weight, prolonged exposure (i.e., >48 h despite negative blood cultures) to empiric antibiotics, and exposure to third-generation cephalosporins and other broadly acting antibiotics such as carbapenems and beta-lactam/beta-lactamase inhibitor combinations [27]. Differences in practices related to these risk factors likely account for the widely varying incidence of Candida infection across centers. Few clinical signs or laboratory assays have been validated for candidiasis in infants, thus prevention of infection is critical, whereas the development and validation of a candida sepsis scoring model, already available for adults [28], in neonates with suspected sepsis could be of great value.

Finally, a variety of viruses (enterovirus, herpes simplex virus, cytomegalovirus, adenovirus, influenza, parainfluenza virus, rhinovirus, coronavirus, respiratory syncytial virus) have been isolated as causative agents of sepsis-like syndromes in neonates as well as nosocomial outbreaks in neonatal intensive care units (NICUs) [26].

## 4. Risk Factors

### 4.1. Maternal Risk Factors

Maternal factors that are associated with higher odds of sepsis in neonates include history of urinary tract infection (UTI) mainly during the third trimester of pregnancy, G.B. *Strep* rectovaginal colonization, G.B. *Strep* bacteriuria, prior birth of infant with G.B. *Strep* infection, *E. coli* perineal colonization, chorioamnionitis/IAI, intrapartum fever, and prolonged (≥18 h) rupture of membranes. Elective C-section, however, performed in the absence of labor or attempts to induce labor, with membrane rupture at the time of surgery and without signs of fetal distress, is associated with a near-zero risk of EOS. Interestingly, primiparous mothers were found to present with higher odds of having a neonate with sepsis than multiparous mothers, which could probably be explained by the prolonged duration of vaginal delivery among primiparous women, which increases the time exposed to a possible pathogen [29,30]. Obstetric practices that interfere with chorioamniotic membranes, disrupting the barrier against ascending infections, are also associated with a higher risk of EOS [10]. Such practices include cervical examinations, membrane sweeping, and operative vaginal birth. Maternal G.B. *Strep* screening and IAP reduce the risk of early-onset sepsis due to G.B. *Strep* but do not eliminate it [18].

### 4.2. Neonatal Risk Factors

Neonatal risk factors include preterm birth, low birth weight, male sex, meconium-stained amniotic fluid, birth by traumatic delivery, perinatal asphyxia, need for resuscitation and/or intubation at birth, and congenital anomalies [10,29,31,32,33]. Other critical risk factors for hospital-acquired infections that provide a portal of entry or impair barrier are central venous catheter use, prolonged intravenous access, subcutaneous indwelling catheters, parenteral nutrition, invasive mechanical ventilation, and prolonged hospitalization [4,34].

Prematurity is the most significant factor correlated with EOS and LOS. In premature infants, immune system immaturity and low concentration of transplacentally acquired maternal IgG antibodies (as this is mainly taking place during the second part of pregnancy), in addition to the prolonged hospital stay that is usually required and its associated numerous risks of infection, predispose this group of infants to infections [2]. Lower neonatal 25-OH vitamin D concentrations have also been associated with EOS [35].

## 5. Clinical Presentation

The initial diagnosis of sepsis is clinical. Therefore, initiation of empiric antibiotic treatment, prior to culture results, is crucial to timely treat sepsis and limit possible complications and adverse outcomes. Clinical identification of sepsis in neonates is very challenging because of the variable, sometimes vague and nonspecific symptoms in this age group. Often, symptoms overlap with other neonatal diseases, such as respiratory distress syndrome (RDS), cyanotic congenital heart disease, meconium aspiration syndrome, congenital causes of bowel obstruction, and metabolic disorders [1]. Symptoms may vary from very subtle to very severe in cases of septic shock. Temperature instability is a common finding in neonates, presenting either as hyperthermia, usually in full-term neonates, or as hypothermia, most often in preterm neonates. In addition, findings from the respiratory system consistent with RDS, including tachypnea, grunting, apnea, cyanosis, nasal flaring, and use of accessory muscles, may occur in EOS [4]. From the cardiovascular system, tachycardia is an early sign of sepsis. In contrast, hypotension and signs of poor perfusion, such as delayed capillary refill, pallor, mottled skin, and cool extremities, tend to arise later. Abnormal heart rates with decreased variability and transient decelerations are found to precede the diagnosis of LOS by hours, even days [36]. Neurologic symptoms include lethargy, irritability, incessant or high-pitched crying, hypotonia, blunt reflexes, poor feeding, and, rarely, seizures. Gastrointestinal signs include feeding intolerance, vomiting, diarrhea, jaundice, abdominal distension, and hepatosplenomegaly. Metabolic dysregulation may consist of hyper- or hypoglycemia, electrolyte disturbances, and metabolic acidosis secondary to poor perfusion. Skin findings include petechiae, purpura, rashes, impetigo, cellulitis, and abscess [1,4].

## 6. Risk Assessment for Sepsis

Nonspecific clinical presentation of neonatal sepsis may delay the recognition and thus the treatment of affected neonates until more ominous clinical signs arise. Therefore, it is of great importance to have risk assessment scores, which help determine early which patients are in need of empiric antibiotics for neonatal sepsis. In this way, neonates who actually need treatment do receive it, and neonates who would be unjustifiably administered antibiotics are spared the potential hazards of their use. Early initiation of antibiotics in septic newborns is vital, as neonatal sepsis can lead to an increased risk of lifelong complications, including neurocognitive sequelae such as cognitive defects, visual and hearing impairments, and cerebral palsy [1]. On the other hand, early and/or prolonged unjustified antibiotic treatment is associated with gut, skin, and respiratory tract dysbiosis and is an independent risk factor for serious adverse events such as necrotizing enterocolitis, subsequent infection (including invasive fungal infections), bronchopulmonary dysplasia, and even death [37].

### 6.1. Risk Assessment for EOS

A significant cause of concern for neonatologists is the decision to initiate antibiotic therapy early in infants who have risk factors for EOS but are in good clinical condition. Another critical issue is when to discontinue antibiotic therapy in neonates with suspected, but not confirmed, sepsis [38]. The American Academy of Pediatrics (AAP) Committee of the Fetus and Newborn and Committee of Infectious Diseases published updated EOS management guidelines in 2018, with two different approaches for the management of neonates born at ≥35 weeks gestational age and neonates born at ≤34, 6/7 weeks gestational age [38,39]. As noted in the AAP document, *“No strategy can be used to immediately identify all infants who will develop EOS or avoid the treatment of a substantial number of infants who are uninfected”* [38]. These strategies contain measures to monitor patients with sepsis not initially identified, as well as neonates without sepsis who will need discontinuation of antibiotic administration.

#### 6.1.1. Neonates ≥ 35, 0/7 Weeks Gestational Age

The AAP Committee on the Fetus and Newborn proposed three potential approaches to EOS risk assessment [38]. Each of the three approaches uses infection risk factors in different ways to identify infants at increased risk of EOS. Each approach has advantages and disadvantages that must be taken into consideration by each center when determining its best practice.

##### Categorical Risk Factor Assessment

This approach relies on the presence or absence of specific risk factor threshold values in order to identify infants at increased risk for EOS. These factors are (1) signs of clinical illness, (2) mother with intrapartum fever and/or clinical diagnosis of intrapartum infection (chorioamnionitis, IAI), (3) indication for G.B. *Strep* IAP, and (4) administration of adequate G.B. *Strep* IAP if indicated. Neonates with the first two risk factors require laboratory testing, including blood culture, and empirical administration of antibiotics. Neonates without risk factors (1) and (2), born by a mother who is colonized with G.B. *Strep*, need routine care if their mother received adequate IAP, but if not, they need to be submitted to laboratory testing if they have additional risk factors, i.e., duration of ROM ≥ 18 h or birth before 37 weeks of gestation.

If there are no additional risk factors, the recommendation is observation for at least 48 h if they develop symptoms [38]. Appropriate IAP is administration of penicillin G, ampicillin, or cefazolin ≥ 4 h before delivery [12]. Any other antibiotic or timing of administration is considered inadequate in this approach. Limitations of this approach include the lack of clear definitions for newborn clinical illness and abnormal laboratory tests, difficulties in establishing the clinical diagnosis of maternal chorioamnionitis, and an inconsistent consideration of intrapartum antibiotics [38]. As a result, this approach overestimates the number of infants who are actually at risk for EOS, and many relatively low-risk neonates are administered empirical antibiotic treatment unnecessarily.

##### Multivariate Risk Assessment (Neonatal Early-Onset Sepsis Calculation)

The first attempt to develop a score for the early recognition of sepsis in newborns was made by U. Töllner and was published in 1982 [40]. Another diagnosis score for EOS was created in 1988, the Hematologic Scoring System (HSS) [41]. The HSS utilizes blood count criteria to assign a score, (leukocyte count, immature/total neutrophil ratio, and platelet count) with increasing sepsis likelihood when the score is ≥3. It is a simple, speedy tool that suffers from lower sensitivity; it is advisable to be used in conjunction to other methods, but its utility is in low-resource clinical settings [42]. The latest efforts focus on multivariate risk assessment.

The multivariate risk assessment considers established risk factors and the clinical condition of the newborn to estimate their risk for EOS. A cohort of 608,000 newborns was used to develop predictive models for culture-confirmed EOS based on objective data known at birth [43] and the evolving newborn condition during 6–12 h post-birth [44]. The objective data include gestational age at birth, highest maternal intrapartum temperature, maternal G.B. *Strep* colonization status, duration of ROM, and type and duration of intrapartum antibiotic therapies. The predictive models were incorporated in a web-based risk calculator known as the Neonatal Early-Onset Sepsis Risk Calculator [45] (https://neonatalsepsiscalculator.kaiserpermanente.org/ (accessed on 15 December 2024)). According to the calculator, neonates with EOS risk estimated at ≥1 per 1000 live births should be clinically observed and investigated with blood culture, while infants with an EOS risk ≥ 3 per 1000 live births should be empirically treated with antibiotics [38]. Several retrospective and prospective studies have demonstrated the utility of the sepsis calculator in substantially reducing laboratory testing and exposure to antimicrobial agents. A prospective validation in 204,685 infants revealed that blood culture testing declined by 66% and empirical antibiotic treatment decreased by 48% with this approach compared to the categorical risk algorithm based on recommendations by the CDC [45]. An advantage of this approach is that it provides an individual infant’s risk using objective data rather than placing the infants in categories with a wide range of risks using ambiguous information. As a result, this approach has decreased the frequency of blood culture testing and unnecessary antibiotic administration compared to the categorical risk assessment, while keeping the number of unidentified infected infants low under the condition that the newborn is closely monitored over the first 48 h after birth.

##### Sepsis Risk Assessment Primarily Based on Newborn Clinical Condition (Enhanced Observation)

This approach relies mainly on the newborn’s clinical condition to identify infants with EOS, regardless of neonatal or maternal risk factors. Infants who appear ill at birth and those who develop signs of illness during 48 h after birth, on clinical observation, have a high risk for EOS and are treated with empiric antibiotics after blood culture is obtained [12,38]. No infant is administered empiric antibiotics if well appearing. Identification of initially well-appearing infants who develop clinical illness is an *anticipated* outcome, not a “*missed case*” or failure of care. This approach is based on multiple studies that have shown that term infants and late preterm infants who appear well at birth have a 60–70% reduction in risk for EOS, so they may be observed in the following 48 h, either with continuous monitoring or with serial examinations, without requiring any further intervention [44,45,46]. Moreover, even in the case of chorioamnionitis-exposed infants, the vast majority of them born at 34–35 weeks’ gestation who were well appearing at birth ultimately did not need antibiotics [47,48]. When comparing this approach to the categorical risk assessment, researchers at one center in Italy (cohort of 7628 term infants managed with categorical risk assessment compared to a cohort of 7611 infants managed with serial physical examinations every 4–6 h through 48 h of age) found that laboratory tests, blood cultures, and the use of empiric antibiotics were significantly reduced in the group of neonates managed with serial physical examinations [49]. Advantages of this approach include lower use of empiric antibiotic treatments compared to categorical approaches. However, centers that adopt this approach must ensure serial, structured, and documented physical assessments and establish clear criteria for additional evaluation and empirical antibiotic treatment.

#### 6.1.2. Neonates ≤ 34, 6/7 Weeks Gestational Age

The strongest predictor of EOS is gestational age [38]. The challenge in this group is the identification of infants at the lowest risk of EOS who could not be started on antibiotics, since the vast majority of this age group presents with some degree of clinical instability. The best approach to achieve this is by assessing the circumstances of preterm birth and recognize low-risk delivery characteristics, thus guiding decisions to limit early antibiotic use [39].

In this context, criteria for preterm infants to be considered at the lowest risk for EOS include all of the following: (1) noninfectious indications for preterm birth, such as maternal preeclampsia, placental insufficiency, and fetal growth restriction, (2) birth by cesarean delivery, and (3) absence of labor, attempts to induce labor, or any ROM before delivery. The approach to these neonates includes either no laboratory investigation and no empirical antibiotics or blood culture and clinical monitoring. With infants that do not show significant clinical improvement after initial stabilization and/or present with severe clinical symptoms, empiric antibiotics may be reasonable but not mandatory [12,39]. Infants in this category born by vaginal or cesarean delivery, after efforts to induce labor and/or ROM before delivery and with no concern for infection during the process of delivery, are managed by obtaining a blood culture and clinical monitoring.

Neonates born preterm because of cervical incompetence, preterm labor, premature preterm rupture of membranes (PPROM), clinical concern for intraamniotic infection/ chorioamnionitis, and/or acute onset of unexplained nonreassuring fetal status are at the highest risk for EOS [12,39]. These infants should undergo EOS evaluation with blood culture and empiric antibiotic treatment. Infants born by induction of labor, with or without cervical ripening and/or rupture of membranes (ROM), before delivery from mothers with indication to receive G.B. *Strep* prophylaxis—who received inadequate G.B. *Strep* IAP—fall into the same category and should be managed accordingly. CSF cultures before the initiation of antibiotics should be obtained in infants whose clinical condition allows such a procedure, without delaying the initiation of antibiotic treatment [39].

Updated AAP guidance for EOS risk assessment among infants ≤ 34 weeks of gestation published in 2018 [39] and updated G.B. *Strep* prevention guidelines published in 2019 [12] recommend the use of low-risk delivery characteristics in order to distinguish low-risk from high-risk preterm neonates and administer empiric antibiotic therapy accordingly. The characteristics of low- and high-risk premature infants for EOS risk assessment are summarized in Table 1.

### 6.2. Other Risk Assessment Tools for Early- and Late-Onset Sepsis

Many efforts have been made to develop scores for the recognition of neonates who are at the highest risk for both EOS and LOS.

Early efforts include the SNAP (Score for Neonatal Acute Physiology) [50], SNAP-II, and SNAPPE-II (Score for Neonatal Acute Physiology with Perinatal Extension) [51] scores for general illness and outlook prediction for neonates. They are widely utilized in NICU settings but are not sepsis-specific per se, although they are utilized as such mostly in formulating treatment strategies for critically ill neonates [52,53,54]. More recent and more specific scores are better for neonatal sepsis [55].

The HeRO Score (Heart Rate Observation) [56] is a non-invasive and continuous monitoring method, providing early indications of sepsis prior to any symptoms by analyzing heart rate deviations. It is useful for both EOS and LOS and is relevant in very low birth weight (VLBW), preterm, and full-term infants in NICUs. Its incorporation with other newer and different rick assessment tools improves the outcome by providing early indications of potential sepsis [57].

A retrospective cohort study in 2022 [58] incorporated thromboelastometry with common neonatal risk factors to create the Neonatal Sepsis Diagnostic (NeoSeD) Score for both term and preterm infants. Its high level of precision in identifying sepsis with an area under the curve (AUC) of 0.918 is impressive, but the use of incorporated thromboelastometry make it useful in developed countries mostly.

In a recent systematic review [59], 16 studies, which included LOS scores using either clinical parameters, laboratory parameters, risk factors, or a combination of the above, were identified. Despite these efforts, no risk score has been widely adopted in clinical practice due to challenges like the lack of a standardized definition of neonatal sepsis, variability in the availability of laboratory tests across settings, and insufficient validation across diverse neonatal populations [59].

For nosocomial sepsis in a NICU setting, and utilizing clinical and laboratory tests such as C-reactive protein, neutrophil fraction, and thrombocytopenia and medical patient history like prolonged parenteral nutrition exposure and fever, the NOSEP (Nosocomial Sepsis) Score [60] could be of predictive value, especially if by a central vascular catheter insertion site and hub colonization are included in the score. This tool is indicative for preterm and VLBW neonates in a NICU setting.

Similarly, but for a low-resource nosocomial environment, the Rosenberg Nosocomial Sepsis Score utilized clinical signs, such as apnea, hepatomegaly, jaundice, lethargy, and pallor to provide a sepsis early diagnosis in preterm neonates [61].

The nSOFA (neonatal Sequential Organ Failure Assessment) score is one proposed tool for quantifying neonatal multi-organ dysfunction and is emerging as a tool to assess mortality risk in preterm infants with sepsis [62]. It utilizes categorical scores in (1) the need for mechanical ventilation and oxygen requirement (score range 0–8), (2) the need for inotropic and/or corticosteroid support for presumed adrenal insufficiency or catecholamine-resistant shock (score range 0–4), and (3) the presence and severity of thrombocytopenia (score range 0–3) [49,50]. Changes in the nSOFA score have been correlated with LOS attributable and all-cause mortality in preterm infants [62,63].

A risk score that includes both blood tests, glucose changes, and CRP levels for early LOS diagnosis in NICU preterm and term neonates is the Sepsis Prediction Score (SPS) presented in 2023 [64]. An SPS score of ≥3 could predict sepsis with 76.60% sensitivity and 72.55% specificity in their prospective study, albeit in a population of 120 retrospectively and 145 prospectively monitored neonates. This is valuable tool in a NICU environment with continuous monitoring.

Another new one is the Phoenix Sepsis Score, a composite four-organ system model comprising criteria for cardiovascular, respiratory, neurological, and coagulation dysfunction. Pediatric sepsis criteria apply to children younger than 18 years and are applicable to newborns or neonates whose postconceptional age is ≥37 weeks. The use of this score could identify potentially life-threatening organ disfunction, predict mortality, and facilitate harmonized data collection on epidemiology of disease globally and in that way support clinical care, quality improvement, and research, thus improving outcomes for neonates and children with sepsis [65]. Table 2 shows details on all the above risk assessment tools. Table 3 presents a neonatal sepsis risk assessment flow and management that suggests all relevant tools in each stage.

Research has been focusing on the rapidly evolving machine learning modeling and artificial intelligence methods, which take readily available individualized patient parameters into account [66] and “omics”-based strategies (metabolomics, proteomics, and genomic approaches), which are promising but not yet ready for clinical application [67].

**Table 2:** Risk assessment tools for neonatal sepsis.

Risk Assesment Tool	Year	Purpose	Factors	Diagnostic Steps	Sepsis Type	Infant Population	Clinical Utility
Töllner Sepsis Score [40]	1982	Early diagnosis of sepsis	CRP, WBC count, clinical signs	Scoring based on infection markers	EOS, LOS	Preterm and term infants	Quick clinical assessment
Hematologic Scoring System (HSS) [41]	1988	Early sepsis detection	Leukocyte counts, I ratio, platelet count	Score ≥ 3 indicates high risk	EOS, LOS	Preterm and term infants	Widely used for early sepsis diagnosis
HeRO Score [56]	2005	Early detection using heart rate variability	Heart rate variability analysis	Continuous monitoring; alerts 24 h before symptoms	EOS, LOS	VLBW, preterm, full term	Early warning system in NICUs
NOSEP Score [60]	2000	Predict nosocomial sepsis	CRP, neutrophil fraction, thrombocytopenia, fever	Uses weighted variables to predict nosocomial sepsis	LOS	NICU neonates	Predicts hospital-acquired infections
Neonatal Early-Onset Sepsis Calculator [45]	2011	Risk stratification for EOS	Maternal fever, G.B. *Strep* status, ROM	Determines need for antibiotics	EOS	Term and preterm infants ≥ 34 weeks GA	Reduces unnecessary antibiotic use
Rosenberg Nosocomial Sepsis Score [61]	2010	Early identification of nosocomial sepsis in low-resource settings	Apnea, hepatomegaly, jaundice, lethargy, pallor	Scoring based on clinical signs	Nosocomial Sepsis	Preterm neonates in low-resource settings	Useful for low-resource NICUs
SNAPPE-II Score [51]	2019	Assess severity in critically ill neonates	Blood pressure, temperature, seizures, urine output	Score calculated within 24 h of NICU admission	EOS, LOS	VLBW, critically ill	Guides treatment decisions
nSOFA Score [63]	2019	Predicts organ dysfunction and mortality	Heart rate, blood pressure, respiratory status	Score > 4 indicates severe risk	LOS	VLBW infants Preterm infants	Mortality prediction in NICUs
NeoSeD Score [58]	2022	High diagnostic accuracy for sepsis	Clinical signs, CRP, liver size, thromboelastometry	AUC ≥ 0.918 indicates sepsis	EOS, LOS	Preterm and term infants	Requires lab-based evaluation
NeoSep Severity & Recovery Scores [68]	2022	Assess severity and monitor recovery	Clinical observations (e.g., feeding, respiratory distress)	Daily assessment for severity and recovery	EOS, LOS	Preterm and term infants	Low-resource settings
Sepsis Prediction Score (SPS) [64]	2023	Early diagnosis of late-onset sepsis	Temperature instability, CRP, platelet count, glucose changes	Score ≥3 indicates sepsis	LOS	NICU neonates	Combines clinical and lab markers
Phoenix Sepsis Score [69]	2024	Predicts sepsis and septic shock	Cardiovascular, respiratory, neurological, coagulation	Score ≥ 2 indicates sepsis severity	EOS, LOS	Full-term infants, childern < 18 yrs old (preterm neonates and term newborns hospitalized directly after birth are excluded)	Useful for comprehensive NICU assessments

CRP: C-reactive protein, WBC: white blood cell, I ratio: immature-to-total ratio, EOS: early-onset sepsis, LOS: late-onset sepsis, VLBW: very low birth weight, NICU: neonatal intensive care unit. G.B. *Strep*: Group B Streptococcus, ROM: rupture of membranes, GA: gestational age, AUC: area under the curve.

**Table 3:** Neonatal sepsis risk assessment and management flow with relevant risk assessment tool suggestions.

Step	Relevant Risk Assesment Systems	Description and Application
1: Initial Assessment	No specific score	Differentiate between: EOS: ≤72 h of life/LOS: >72 h Guides subsequent steps in risk assessment
2: Evaluate Clinical Signs and Risk Factors	Categorical Risk Factors, Neonatal Sepsis Calculator	Assess maternal, neonatal, and environmental factors: - Maternal fever, prolonged rupture of membranes (ROM) - Respiratory distress, poor feeding Use categorical risk factors for EOS and LOS or the Neonatal Sepsis Calculator for personalized risk estimation
3: Calculate Risk Score	nSOFA, Phoenix Score, NeoSeD Model, HSS, NOSEP Score, SNAPPE-II, HeRO Score	Assign points based on severity of clinical instability: nSOFA: Evaluates multi-organ dysfunction, especially in VLBW infants Phoenix Score: Focuses on cardiovascular, respiratory, neurological, and coagulation systems (USE ONLY in full-term and childern < 18 yrs old and non-hospital-accuired sepsis patients) NeoSeD Model: Integrates clinical signs, CRP levels, and thromboelastometry HSS: Uses blood markers like leukocyte counts NOSEP Score: Focuses on detecting nosocomial infections in NICUs SNAPPE-II: Measures illness severity using physiological and perinatal HeRO Score: Uses heart rate variability for early sepsis detection
4: Risk-Based Management	Neonatal Sepsis Calculator, Enhanced Observation	Low Risk: Monitor closely; no immediate intervention Moderate Risk: Lab tests; consider antibiotics High Risk: Immediate empirical antibiotics and full workup
5: Ongoing Monitoring and Adjustment	nSOFA, Enhanced Observation, HeRO Score	Continuous reassessment of clinical status: nSOFA: Monitors changes in multi-organ dysfunction. HeRO Score: Provides continuous monitoring using heart rate variability for early sepsis warnings. Enhanced observation for well-appearing neonates to avoid unnecessary interventions. Adjust antibiotics based on culture results and clinical improvement.
6: Special Considerations for Preterm Infants	NeoSeD, NOSEP, SNAPPE-II, Multivariate Risk Assessment	Use the NeoSeD Model for preterm neonates with clinical instability. NOSEP Score: Targets nosocomial infections, especially in VLBW neonates. SNAPPE-II: Provides comprehensive assessment of critically ill neonates. Multivariate risk assessment for neonates ≥34 weeks using the Neonatal Sepsis Calculator to guide antibiotic use.

CRP: C-reactive protein, EOS: early-onset sepsis, LOS: late-onset sepsis, VLBW: very low birth weight, NICU: neonatal intensive care unit.

### 6.3. Sepsis Clinical Pathways

There are several prominent institutes and their NICU clinics that have moved on from utilizing single tools and have updated their neonatal risk assessments and turned them into clinical pathways [70,71] that incorporate several characteristics from all the above risk tools without explicitly using their scoring systems. There is no citing literature to our knowledge for these, and they are not validated beyond the utilization/interpretation of national guidelines [71]. Not all are uploaded on their respective websites, so we only mention the most prominent ones that also have their pathways publicly available. There are several in the US and Australia.

The Children’s Hospital of Philadelphia (CHOP) in the US provides an N/IICU Clinical Pathway for Evaluation and Treatment of Suspected Sepsis in Newborns and Infants that splits strategies into zones pending differential evaluation, mostly using the principles of nSOFA and HSS without directly naming them [72]. Similarly, the Johns Hopkins All Children’s Hospital Pediatric Sepsis Clinical Pathway incorporates comprehensive assessments from clinical signs incorporating laboratory and cardiological data, consistent with the nSOFA and HeRO tools [73].

Oregon Health & Science University (OHSU) has a very detailed Neonatal Fever/Suspected Sepsis Pathway [74] that utilizes clinical symptoms. In a similar manner, The Royal Children’s Hospital Melbourne provides a sepsis assessment and management guideline that is endorse by the Paediatric Improvement Collaboration in Australia and uses a combination of clinical signs and laboratory testing, with similarities to the nSOFA and NeoSeD approaches, again without explicit mention [75].

The only clinical pathway that is so far validated in a clinical study is from the Children’s Hospital Colorado Emergency Care Network [76]. It is a two-tiered pathway focusing on severe and possible sepsis using differentiated care protocols offering a significant enhancement in neonatal sepsis management. It was implemented across six pediatric emergency departments with two pathways: the Sepsis Stat pathway for confirmed severe cases and Sepsis Yellow for uncertain cases for close monitoring and evaluation before administering antibiotics. Its main aim was to reduce time to antibiotics and intensive care admissions. The study found substantial improvements in median time to antibiotics, down to only 43 min in severe cases, and showed a reduction in NICU admissions from 45% to 34%. In the Sepsis Yellow pathway, a sizable 23% of patients were successfully treated and discharged without antibiotic therapy, emphasizing its effectiveness as a pathway [76].

These protocols are mostly tailored to specific institutional practices and resources that are geographically and epidemiologically distinct and thus cannot be directly assimilated into practice in another world location, but they do demonstrate the broad applicability of established sepsis evaluation principles. They are worth knowing and can provide a basis for localized versions and strategies but do not negate the need for proper validation and documentation, with a notable exception [76]. Table 4 presents examples of neonatal sepsis clinical pathways from reputable institutions worldwide, along with their web links.

## 7. Laboratory Testing

### 7.1. Microbiological Culture Methods

A positive culture from a normally sterile body fluid (blood, CSF, urine), in the context of an analogous clinical picture, verifies the diagnosis of neonatal sepsis

#### 7.1.1. Blood Culture

Modern, computer-assisted automated blood culture systems use optimized enriched culture media, provide continuous-read detection, and identify up to 95% of all microorganisms by 36–48 h of incubation. In neonates, however, the sensitivity of blood cultures may be decreased due to maternal intrapartum antibiotic administration, organisms such as anaerobes that are difficult to grow, small sample volumes, and low-level bacteremia [77]. The delay in pathogen identification and subsequent antibiotic susceptibility testing increases exposure to broad-spectrum antibiotics, which, among other adverse consequences, increases the risk for bacterial antibiotic resistance [77]. The most important factor for identifying a pathogen in a blood culture is the volume of blood samples collected. In routine practice, a culture is obtained in an aerobic bottle. Pediatric blood culture bottles require a minimum volume of 1 mL of blood for optimal detection of microorganisms, even in the presence of very low bacteremia (1–10 CFU/mL) [78]. However, using two separate culture bottles (one aerobic and one anaerobic) may be helpful in differentiating skin commensals from true infections. Moreover, most neonatal aerobic pathogens, such as G.B. *Strep*, *E. coli*, and *S. aureus*, will grow under anaerobic conditions. In the presence of a central venous catheter (CVC), two blood cultures (one from CVC and one peripheral) are recommended.

#### 7.1.2. CSF Culture

Some microorganisms might be detected only in CSF and not in the blood. Meningitis incidence ranges from 6% to 12% in EOS and 1% to 12% in LOS, depending on gestational age and isolated pathogen [5,79]. In EOS, lumbar puncture (LP) should ideally be performed on all symptomatic newborns who are at the highest risk along with blood culture before the initiation of empiric antibiotics, provided that the procedure is feasible, does not compromise the infant’s clinical condition, and does not significantly delay treatment initiation [38]. In case a CSF culture is not obtained and the blood culture is positive, or the clinical course and laboratory data of the patient strongly suggest bacterial infection, CSF culture and analysis need to be performed to optimize the type and duration of antibiotic therapy. An LP is also indicated when the infant is not improving with appropriate antimicrobial therapy or when there are clinical signs from the central nervous system, such as apnea, lethargy, and seizures [80]. In LOS, LP should be part of the routine evaluation, since meningitis could happen without sepsis, especially in VLBW infants [81]. Moreover, some LOS cases with bacteremia may be complicated by meningitis [81].

#### 7.1.3. Urine Culture

Urine cultures are not routinely obtained in neonates with EOS unless there is a suspected congenital urogenital anomaly, but they must be obtained in infants with LOS [80]. Urine specimens could be obtained by suprapubic bladder aspiration or urinary catheterization.

### 7.2. Biomarkers

#### 7.2.1. Complete Blood Count

A complete blood count is an additional test commonly used to evaluate neonates for sepsis. White blood cell (WBC) count varies during the first 12 h of life, so serial WBC counts obtained several hours apart may be more helpful. Leukopenia (WBC count < 5000/mm^3^) has a better predictive value than leukocytosis (WBCs > 40,000–50,000/mm^3^), but a normal leukocyte count does not rule out sepsis.

Absolute neutrophilic count < 1000/mm^3^ at ≥6 h of age may be a significant finding with an ominous prognosis, especially in EOS. Immature to total neutrophil ratio (I:T) > 0.22 for preterm and >0.27 for term neonates is more specific and has good predictive value if present [1,82]. However, the I:T ratio may be elevated in 25–50% of uninfected infants [80]. Thrombocytopenia is a frequent finding in neonatal sepsis but is nonspecific and appears as a late symptom.

#### 7.2.2. Blood Coagulation Tests

Recent evidence highlights the need to carefully monitor clotting parameters during neonatal sepsis. Aziz et al. (2024) [83] reported that coagulopathy evaluations in the NICU often coincided with infection by Gram-negative pathogens, higher nSOFA scores, and worse outcomes. Notably, a striking 80% of these evaluations revealed abnormal coagulation, most commonly prolonged prothrombin times. In parallel, another study [84] observed a progressive shift toward hypercoagulation in preterm neonates with Gram-positive sepsis, coupled with increased glycoprotein expression. Taken together, these findings emphasize the crucial role of coagulation profiling as both a biomarker and therapeutic target.

#### 7.2.3. Acute Phase Proteins

Single values of C-reactive protein (CRP) and procalcitonin after birth should not be used as a guide for decision-making regarding the initiation or discontinuation of antibiotic treatment, since their values in neonates may be elevated for various noninfectious reasons, including fetal hypoxia, delivery stress, RDS, meconium aspiration, IVH, surgery, and pneumothorax [85,86]. Each has limited and inconsistent sensitivity and specificity, so neither is currently recommended as a single marker of infection. CRP begins to rise between 8–12 h and peaks at 24–48 h after infection onset [85,86,87], so, due to its delayed rise as a response to infection, CRP has an unacceptable low sensitivity within the first 24 h for the early diagnosis of neonatal sepsis.

Procalcitonin has a slightly superior sensitivity than CRP at the beginning of the infection, since it rises rapidly in 2–4 h and peaks at around 6–12 h. However, its specificity is slightly lower [78,87,88,89]. Serial measurements of CRP at 24–48 h after onset of symptoms have increased sensitivity and negative predictive value and may be helpful in monitoring the response to treatment and guiding the antibiotic therapy [80,83,88]. Two normal CRP values obtained 8–24 h after birth and 24 h later have a negative predictive value of 99.7% for proven neonatal sepsis and are helpful for discontinuing antibiotics [80,89]. CRP and PCT are better indicators of sepsis when serial measurements are taken and/or a combination of biomarkers are taken into account with a correlation of their values and trends with clinical findings [90].

#### 7.2.4. Other Markers of Inflammation

Other markers of inflammation, such as serum amyloid A (SAA), interleukins (IL-6, IL-8, sIL-2 receptor), and tumor necrosis factor α (TNF-α), are addressed in several studies as “early warning biomarkers” of neonatal sepsis, especially if they are combined with CRP but are not routinely used in clinical practice [87,88,91,92]. Neutrophil surface antigens CD11, CD14, CD64, and sCD14 (presepsin) are promising early infection markers that correlate well with CRP. Presepsin has been shown in meta-analyses to be as accurate as procalcitonin (PCT) and C-reactive protein (CRP) in diagnosing neonatal sepsis. Presepsin levels rise after 2 h, i.e., earlier than procalcitonin, with a peak at 3 h after infection onset and decline after 4–8 h [93]. It can be used to monitor treatment with antibiotics and show their effectiveness. It is a reliable tool for the early diagnosis of Gram-negative and Gram-positive sepsis and fungal infections [94]. Most factors affecting CRP and procalcitonin levels do not affect presepsin levels, such as mode of delivery, maternal fever, and stained amniotic fluid, which suggests that presepsin could become an effective tool for discriminating between infectious and non-infectious inflammatory conditions [95]. In comparison with other biomarkers, presepsin demonstrated the highest accuracy, with 88.9% sensitivity and 85.7% specificity in diagnosing early-onset sepsis [95].

### 7.3. New and Molecular Techniques

Polymerase chain reaction (PCR) is a highly sensitive and rapid technique that is increasingly being applied to body fluids directly without the need to first culture causative agents. Furthermore, various diagnostic systems have been developed to expedite organism identification in positive blood cultures, offering faster results compared to conventional methods. FDA-cleared assays, such as Peptide Nucleic Acid Fluorescent In Situ Hybridization Molecular Stains (PNA-FISH), Matrix-assisted laser desorption–ionization/time-of-flight (MALDI-TOF), and PCR-based methods, e.g., GeneXpert (quantitative PCR, qPCR), FilmArray (multiplex PCR), and Verigene (combination of microarray technology and PCR), contribute to swift identification, with some providing results in as little as 20 min [96]. In particular, qPCR, apart from the identification of the pathogen, also quantifies the amount of DNA present in a sample, providing information about infection severity. It also demonstrates a high negative predictive value while requiring only a small sample volume. Disadvantages of qPCR include the inability to conduct susceptibility testing, a high sensitivity that does not differentiate between active infection or recent infections that have been resolved, and the high possibility of detecting concomitants, so clinical correlation with the results is necessary [97]. Multiplex PCR is particularly valuable in polymicrobial infections, because it allows for detecting various pathogens in a single test, providing a more comprehensive assessment [96]. These rapid assays have shown promising results in enhancing clinical outcomes, reducing hospital stays, and cutting healthcare costs. Other molecular techniques allow direct amplification of microbial pathogens from whole blood samples within 12 h (such as SeptiFast, SepsiTest, and T2 Magnetic Resonance), detecting amplified pathogen DNA with varying pathogen coverage and turnaround times [96].

Despite the potential of molecular diagnostics as adjunctive tests in neonatal sepsis diagnosis, challenges like discordance with culture methods, false positives, cost, and availability limit their readiness to replace blood cultures as reference standards [1,78]. Omics technology and machine learning are dynamic evolving methods which may provide us with personalized diagnostic and prognostic models for the future [78].

## 8. Management

Neonatal sepsis management focuses on maintaining hemodynamic stability and providing supportive care for potential organ dysfunction along with targeting the cause of the disease. Intravenous fluids, volume expanders (such as normal saline), inotropes, oxygen support, antibiotics, antiviral drugs, and antifungal medications are used for this purpose. The distinct causes and microorganisms involved in EOS and LOS lead to various treatment approaches. Choosing between empirical or targeted treatment hinges on factors like sepsis type, infection origin (nosocomial or community-acquired), health condition and comorbidities of the patient, and, most importantly, the isolation of the causative pathogen in the cultures obtained. Factors such as PROM, stained amniotic fluid, and vaginal colonization also influence treatment decisions, aiding in selecting the most appropriate antimicrobial therapy for the patient.

Once blood cultures are negative within 36–48 h of incubation, therapy cessation is recommended, unless there is evidence of site-specific infection or signs of clinical illness [38,39]. Otherwise, the duration of treatment depends on the type of infection (e.g., bacteremia, meningitis), the causative agent (bacteria, fungi, viruses), and the response to treatment. Certain experts propose that a 10-day course is sufficient in most cases, extending up from two to three weeks or even more in some Gram-negative and fungal infections of the blood or CSF [1]. Currently, however, there remains no consensus regarding the ideal duration of antibiotic treatment in neonatal sepsis [1,38,39]. Monitoring antibiotic toxicity is important, especially in renal and/or hepatic failure cases.

On that front, Switzerland has very recently updated the national guidelines of the Swiss Society of Neonatology and the Pediatric Infectious Disease Group Switzerland on early-onset sepsis risk to urge for safe reduction of unnecessary antibiotic use [98]. They propose a probabilistic management due to the continuous decline of sepsis incidences in high-income countries. Thus, they do not recommend the use of an EOS calculator for sepsis since they believe that it aids antibiotics overuse in non-necessary cases, and they recommend serial observations more in low-risk neonates [98].

## 9. Antibiotic Treatment

### 9.1. Antibiotic Treatment of EOS

The predominant causes of EOS in both preterm and term neonates are Group B *Streptococcus* (G.B. *Strep*) and *E. coli*. Initial empiric treatment for suspected EOS typically is a combination of ampicillin and an aminoglycoside, such as gentamicin, to cover for common pathogens, including G.B. *Strep*, *streptococci* spp., *enterococci* spp., and *L. monocytogenes*. The AAP guidance for both term and preterm infants continues to recommend this combination as the most appropriate empiric regimen for those with suspected sepsis [38,39]. Although resistant strains are uncommon, a high proportion of *E. coli* is resistant to ampicillin, and evidence of aminoglycoside resistance is growing [99]. Third-generation cephalosporins should be avoided as an empirical treatment because they are associated with increased risk for antibiotic resistance and invasive fungal infections.

Despite the fact that broader-spectrum antibiotics are not routinely recommended due to overall low resistance rates, they may be considered for critically ill newborns who deteriorate despite being on ampicillin and gentamicin or in infants at the highest risk for Gram-negative EOS, such as preterm infants born after PROM and infants exposed to prolonged antepartum maternal antibiotic treatment, until culture results are available [38,39]. Adding a third- or fourth-generation cephalosporin or a carbapenem to ampicillin and gentamicin may be appropriate in such cases. If Gram-negative meningitis is suspected, a third- or fourth-generation cephalosporin should be added to the regimen. Βeta-lactam antistaphylococcal antibiotics (e.g., nafcillin) are advised if there is suspicion of a Gram-positive organism, such as *staphylococci* spp., with vancomycin being reserved for methicillin-resistant *staphylococcus aureus* (MRSA), coagulase-negative *staphylococci*, ampicillin-resistant *streptococci*, or *enterococci* spp. cases. If there is a suspicion of a fungal infection, it is recommended to initiate empirical treatment with amphotericin.

Definitive treatment should use the narrowest spectrum of appropriate antibiotics, guided by official expert guidelines, culture results, and sensitivity results. Continuous monitoring and antibiotic adjustments should align with culture and CSF analysis, as well as infectious disease consultation in complex or resistant cases.

### 9.2. Antibiotic Treatment of LOS

LOS commonly involves Gram-positive organisms, mainly coagulase-negative staphylococci (CoNS). *Staphylococcus aureus*, Gram-negative bacteria, fungi (mainly *Candida* spp.), and viruses (HSV, enterovirus, rhinovirus, RSV, CMV) are also implicated. Although there is no consensus upon the ideal empirical antibiotic regimen for LOS [15], initial treatment for community-acquired sepsis typically includes a beta-lactam (usually ampicillin) with an aminoglycoside (usually gentamicin). Cefotaxime is added when there is a concern for meningitis (ceftriaxone should be avoided due to potential complications such as hyperbilirubinemia and the formation of calcium–ceftriaxone crystals). In the setting of a nosocomial sepsis, given the high rates of LOS due to CoNS (often resistant to beta-lactam antibiotics), vancomycin (or an antistaphylococcal beta-lactam antibiotic such as nafcillin) combined with an aminoglycoside (gentamicin or amikacin) may be a reasonable choice [15]. Clindamycin or metronidazole may be added for anaerobic coverage in necrotizing enterocolitis cases [15].

Gram-negative pathogens (such as *Enterobacter*, *Klebsiella*, *Serratia*, and *Pseudomonas* spp.) usually require a combination of a beta-lactam with a beta-lactamase inhibitor (such as piperacillin–tazobactam) and an aminoglycoside. In infections by extended-spectrum beta-lactamase-producing pathogens, a carbapenem (such as meropenem) is recommended.

If *Candida* spp. are suspected, empirical antifungal therapy with amphotericin B deoxycholate is recommended as first line [100]. In NICUs with high rates of invasive candidiasis (i.e., >5–10%), antifungal prevention measures involve limiting central catheter use, proper hand hygiene, antibiotic stewardship, and prophylactically fluconazole in high-risk infants (VLBW with central catheters).

As with any antibiotic treatment, the selection is often refined based on the pathogen isolated from the culture results, the timing of obtaining a sterile culture, the clinical condition of the neonate, and the response to therapy. Consulting with infectious disease specialists is often beneficial, especially in complicated or resistant cases of LOS.

## 10. Antiviral Therapy

Antiviral therapies are available for newborns infected with HSV (acyclovir), RSV (ribavirin), CMV (ganciclovir or valganciclovir), and influenza A and B (oseltamivir) [101]. Because early use of acyclovir for herpes simplex infections in neonates has been associated with improved outcomes, physicians may choose to begin therapy for presumptive HSV disease and reevaluate when information on clinical course and results of cultures and PCR assay become available [102].

## 11. Complications

Neonatal sepsis has profound implications on both short-term and long-term outcomes, influencing mortality, neurological development, and physical growth, particularly in preterm infants [1]. Death due to neonatal sepsis is inversely related to gestational age, while Gram-negative sepsis and fungal infections are associated with higher rates of mortality [18,103]. Bakhuizen et al. conducted a meta-analysis of studies assessing short-term and long-term outcomes of neonatal sepsis, revealing that neonates affected by sepsis demonstrated an increased incidence of RDS, intraventricular hemorrhage (IVH), and periventricular leukomalacia (PVL) [104]. The neonatal mortality rate was significantly elevated in sepsis cases (30.9%) compared to the control group (17%). Furthermore, this study underscored the enduring consequences of neonatal sepsis, associating it with increased post-discharge mortality (RR: 2.1) and an increased risk of bronchopulmonary dysplasia.

Additional complications outlined in the literature are pulmonary hypertension, cardiac failure, shock, renal failure, cerebral infarction, retinopathy of prematurity, adrenal hemorrhage and/or insufficiency, bone marrow dysfunction (neutropenia, thrombocytopenia, anemia), and disseminated intravascular coagulation [2]. Meningitis in neonates may result in complications, such as abscess formation, ventriculitis, septic infarcts, hydrocephalus, and subdural effusions [2], with serious long-term adverse neurodevelopmental outcomes, such as cerebral palsy, cognitive impairment, and psychomotor delay. Visual and auditory impairments are also possible complications of neonatal sepsis and/or meningitis [10,104]. Regarding physical development, extremely low birth weight neonates with sepsis exhibited lower weight and height along with impaired head growth during follow-up visits at the corrected age of two years [105].

## 12. Prevention

Prevention strategies are key to reduce the incidence of neonatal sepsis. Very important for EOS prevention is universal screening of pregnant women at 36, 0/7 to 37, 6/7 weeks of gestation for vaginal and rectal G.B. *Strep* colonization and appropriate targeted IAP at the time of labor or rupture of membranes, when indicated [12,106,107,108]. In addition, in case of threatened preterm labor and preterm prelabor rupture of membranes (PPROM), women should be screened for G.B. *Strep* colonization on admission unless a G.B. *Strep* culture was obtained within the preceding 5 weeks and should receive G.B. *Strep* prophylaxis unless the screening results are negative [12,106,107,108]. If certain risk factors are present, i.e., PROM (>18 h before delivery), maternal fever (>38 °C during labor), or foul-smelling amniotic fluid, WHO recommends empirical antibiotics for neonates until cultures are negative at 36–48 h, especially in settings in which referral is not possible [109].

Early initiation of enteral feeds, minimal enteral feeding as soon as possible after birth (ideally in the first hours of life in preterm neonates), and promoting the use of breast milk and breast feeding are all measures of great importance in sepsis prevention [110,111]; however, the latest meta-analysis of the few clinical trials did not show benefits on necrotizing enterocolitis and sepsis [112,113]. These practices minimize the duration of parenteral feeding and contribute to acquisition of a healthy microbiome of the neonate [110,111]. Other interventions such as kangaroo mother care (ideally immediately after birth and daily thereafter) and other thermal care practices contribute to the reduction in neonatal mortality due to infections [114,115].

Stewardship of antibiotics and use with caution steroids (pre- and postnatal administration) and proton pump inhibitors (PPIs) are of great importance as well [116,117,118]. In NICUs with high rates of invasive candidiasis (i.e., >5–10%), antifungal prevention measures involve limiting central catheter use, proper hand hygiene, antibiotic stewardship, and antifungal prophylaxis with fluconazole in high-risk infants, i.e., VLBW with central catheters, and those receiving prolonged parenteral nutrition or antibiotic therapy. These practices are associated with a significant reduction in invasive fungal infections [116,119,120].

Maternal vaccination during pregnancy against diseases that can cause sepsis (such as influenza, Bordetella pertussis, and RSV) can also offer protection via placental transfer of maternally derived antibodies [121,122]. Prenatal counseling of the mothers for specific dietary precautions regarding *Listeria* can also contribute to prevent the transmission of *Listeria*. Screening and management of maternal infections during pregnancy, such as syphilis, human immunodeficiency virus, herpes simplex virus, and hepatitis B virus, is of great importance as well to preventing vertical transmission.

For the prevention of LOS, minimization of invasive procedures, hand hygiene, antiseptic measures, and adherence to infection control protocols for catheter insertion and maintenance (NICU central catheter bundles) are crucial [119]. Hand hygiene, in particular, is the single most important strategy for preventing hospital-acquired infections in NICUs, an inexpensive and cost-effective method of such great importance [123].

Under research are vaccines and synthetic monoclonal antibodies toward specific pathogens of neonatal sepsis, such as G.B. *Strep* [124]. Immunomodulatory therapies in neutropenic infants (such as intravenous immunoglobulin—IVIG, granulocyte colony-stimulating factor—G-CSF, and granulocyte–macrophage colony-stimulating factor—GM-CSF) and inflammatory inhibitors (endotoxin inhibitors, cytokine inhibitors, nitric oxide synthetase inhibitors, and neutrophil adhesion inhibitors) are being evaluated for their possible role in the prevention of sepsis but are not yet recommended for routine use in neonates [125,126].

Finally, probiotics, prebiotics, and lactoferrin are being researched for their possible use in preventing LOS and necrotizing enterocolitis [127]. Routine probiotic supplementation demonstrates substantial benefits for preterm infants with reduction to all-cause mortality, necrotizing enterocolitis, LOS, and feed intolerance [128,129].

## 13. Conclusions

Neonatal sepsis, a significant contributor to neonatal morbidity and mortality worldwide, necessitates an in-depth understanding of its diverse etiologies, modes of transmission, and varying clinical presentations across early- and late-onset cases of term and preterm infants. Addressing the ambiguity in defining sepsis stands as a critical step toward unified interpretation and appropriate patient management. Understanding the distinct risk factors and adopting appropriate sepsis risk factor assessment tools and sepsis scores in clinical practice could contribute substantially to the early identification of a neonate with sepsis, which is of pivotal importance for prompt antibiotic treatment and, consequently, better short- and long-term outcomes for the neonates with sepsis. On the other hand, effective preventive strategies, encompassing measures from prenatal care to infection control in healthcare settings, could also play a crucial role in reducing the incidence of neonatal sepsis.

Evolving laboratory diagnostic methods, such as molecular techniques and new inflammatory biomarkers, offer promise for more accurate and rapid detection of disease, essential for guiding targeted treatment, in order to mitigate the severity of neonatal sepsis and thus its short- and long-term consequences. Continued research into novel preventive and therapeutic interventions, alongside established practices like immunization and stringent infection control measures, promises to further reduce neonatal sepsis’s burden and improve outcomes.

## Figures and Tables

**Table 1:** Early-onset sepsis risk assessment of premature infants (≤34 6/7 weeks’ GA).

Low-Risk Preterm Infants	High-Risk Preterm Infants
Maternal preeclampsia	Cervical incompetence
Placental insufficiency	Preterm labor
Fetal growth restriction	PPROM
Cesarean delivery	Intraamniotic infection/chorioamnionitis
Absence of labor or attempts to induce labor	Acute onset of unexplained nonreassuring fetal status
No ROM (rupture of membranes) before delivery	

GA: gestational age, ROM: rupture of membranes, PPROM: preterm premature rupture of membranes.

**Table 4:** Examples of neonatal sepsis clinical pathways from reputable institutions across different regions, along with their web links.

Institution	Pathway Name	Description	Web Link
Children’s Hospital of Philadelphia (CHOP) [72]	Suspected Sepsis Clinical Pathway—N/IICU	Provides guidelines for evaluating and treating suspected sepsis in newborns and infants in the Neonatal/Infant Intensive Care Unit.	CHOP Sepsis Pathway
Johns Hopkins Medicine [73]	Pediatric Sepsis Clinical Pathway	Offers a comprehensive approach to the recognition and management of pediatric sepsis, including neonatal considerations.	Johns Hopkins Sepsis Pathway
Oregon Health & Science University (OHSU) [74]	Neonatal Fever/Suspected Sepsis Clinical Pathway	Outlines the evaluation and management of infants ≤ 28 days old presenting with fever or suspected sepsis.	OHSU Sepsis Pathway
Royal Children’s Hospital Melbourne [75]	Sepsis—Assessment and Management	Provides guidelines for the assessment and management of sepsis in pediatric patients, including neonates.	RCH Melbourne Sepsis Pathway
Children’s Hospital Colorado Emergency Care Network [76]	Two-Tiered Sepsis Pathway	Implements a two-tiered system (Sepsis Stat and Sepsis Yellow) to improve sepsis management, reduce ICU admissions, and promote antibiotic stewardship.	Not available

## Data Availability

Not applicable.
